# Efficient Inverted Solar Cells Using Benzotriazole-Based Small Molecule and Polymers

**DOI:** 10.3390/polym13030393

**Published:** 2021-01-27

**Authors:** Ja Eun Lee, Yoon Kim, Yang Ho Na, Nam Seob Baek, Jae Woong Jung, Yura Choi, Namchul Cho, Tae-Dong Kim

**Affiliations:** 1 Department of Advanced Materials, Hannam University, Daejeon 34054, Korea; 20194142@gm.hannam.ac.kr (J.E.L.); rladbs5334@gmail.com (Y.K.); yhna@hnu.kr (Y.H.N.); 2 ChemOptics, Daejeon 34026, Korea; bnbn3237@gmail.com; 3 Advanced Materials Engineering for Information and Electronics, Kyung Hee University, Youngin 17104, Korea; wodndwjd@khu.ac.kr; 4 Department of Energy Systems Engineering, Soonchunhyang University, Asan 31538, Korea; bnbn3237@sch.ac.kr

**Keywords:** polymer solar cells, small-molecule solar cells, morphology, benzotriazole, molecular aggregation

## Abstract

We synthesized medium-band-gap donor-acceptor (D-A) -type conjugated polymers (PBTZCZ-L and PBTZCZ-H) consisting of a benzotriazole building block as an acceptor and a carbazole unit as a donor. In comparison with the polymers, a small conjugated molecule (BTZCZ-2) was developed, and its structural, thermal, optical, and photovoltaic properties were investigated. The power conversion efficiency (PCE) of the BTZCZ-2-based solar cell devices was less than 0.5%, considerably lower than those of polymer-based devices with conventional device structures. However, inverted solar cell devices configured with glass/ITO/ZnO:PEIE/BTZCZ-2:PC_71_BM/MoO_3_/Ag showed a tremendously improved efficiency (PCE: 5.05%, *J*_sc_: 9.95 mA/cm^2^, *V*_oc_: 0.89 V, and FF: 57.0%). We believe that this is attributed to high energy transfer and excellent film morphologies.

## 1. Introduction

Recently, π-conjugated small molecules and polymers have attracted considerable attention owing to their light weight, good mechanical flexibility, and compatibility with roll-to-roll printing processes that are potentially applicable to solution-fabricated electronic and optoelectronic devices [1,2,3,4,5]. In contrast to polymers, the production of π-conjugated small molecules can be easily scaled-up and high-purity molecules can be obtained [6,7,8]. In addition, they exhibit good crystallinity and a well-defined chemical structure [9]. Using suitable design strategies, π-conjugated small molecules can be adapted to control molecular orbital energy levels and optical transitions, thus achieving desired properties for specific device functions [10]. For example, π-conjugated small molecules with acceptor-donor-acceptor (A-D-A) structures are promising materials for bulk heterojunction organic photovoltaics (OPVs). The power conversion efficiency (PCE) of 11.7% has been obtained for such small molecules by optimizing their molecular structure and device fabrication conditions using an open-circuit voltage (*V_oc_*) of 0.95 V, short-circuit current (*J_sc_*) of 15.7 mA/cm^−2^, and fill factor (FF) of 76% [11]. It is known that π-conjugated small molecules possessing a medium optical energy bandgap (E_g_) exhibit high PCEs owing to the complementary broad absorption of fullerene acceptors (PC_71_BM) [12]. Recently, Zhu et al. reported a remarkable PCE of 13.7% of a binary π-conjugated small molecular system of BSFTR as a donor and Y6 as an acceptor [13]. 

The units of benzotriazole (BTZ) and its derivatives are relatively weak electron acceptors with medium-level E_g_ compared to those of benzothiadiazole, diketopyrrolopyrrole, and quinoxaline building blocks [14,15]. BTZ molecules can act as efficient light absorbing materials in solar cell devices because their molecular structure can prevent aggregation, despite the disadvantage of a wide band gap [16]. In addition, the solubility of BTZ molecules can be improved by directly linking alkyl chains on nitrogen atoms while maintaining a planar conformation [17]. When electron-withdrawing fluorine atoms are introduced into BTZ building blocks, their derivatives can be utilized as efficient acceptor alternatives to fullerenes [18,19,20]. We recently reported BTZ-based polymers with a medium E_g_ of 2.04 eV, highest occupied molecular orbital of −5.54 eV, and lowest unoccupied molecular orbital of −3.50 eV. The highest PCE of 3.81% was achieved with a *V_oc_* of 0.88 V, *J_sc_* of 7.69 mA/cm^2^, and FF of 55.9% when used in inverted solar cells with a ZnO interlayer [21].

In this work, newly developed π-conjugated small molecules (**BTZCZ-2**) and polymers containing BTZ building blocks with carbazole units as donor materials were synthesized and characterized for photovoltaic applications. Polymerization was carefully controlled to obtain a low-molecular-weight polymer (**PBTZCZ-L**) and a high-molecular-weight polymer (**PBTZCZ-H**). The effects of molecular weights on OPV performance were investigated with respect to the structural characteristics. Among the three materials, the **PBTZCZ-H**-based device exhibited the best device performance, with *J*_sc_ of 11.5 mA/cm^2^, *V*_oc_ of 0.74 V, FF of 0.60, and PCE of 5.13%. Surprisingly, the **BTZCZ-2** based device, which shows a wider bandwidth compared to those of the BTZ polymers, possessed a high PCE of 5.05% with *J*_sc_ of 9.95 mA/cm^2^, *V*_oc_ of 0.89 V, and FF of 0.57. 

## 2. Experimental Details

**Materials and characterization** 4-(5-Bromothiophen-2-yl)-2-octyl-7-(thiophene-2-yl)-2H-benzo[d][1,2,3]triazole (**1**) [22], 4,7-bis(5-bromothiophen-2-yl)-2-octyl-2H-benzo[d][1,2,3] triazole (**2**) [23], and 9-(heptadecan-9-yl)-2,7-bis(4,4,5,5,-tetramethyl-1,3,2-dioxaborolan-2-yl)-9H-carbazole (**3**) [24] were synthesized according to the literature. For structural characterization, a Varian 300 spectrometer and Perkin-Elmer spectrophotometer were used to record ^1^H and ^13^C NMR spectra (300 MHz) and UV/vis spectra, respectively. Differential scanning calorimetry (DSC) and thermogravimetric analysis (TGA) were performed using TA Instruments Q50 at a ramping rate of 10 °C/min under N_2_ atmosphere. Molecular weight and polydispersity index (PDI) were measured using a Waters 1515 gel permeation chromatograph (GPC) with a refractive index detector at room temperature (RT) with chlorobenzene as the eluent and polystyrene as the standard. Atomic force microscopy (AFM) was performed using a Digital Instruments Nanoscope IV operated in tapping mode (~350 kHz frequency, Si tip).

**BTZCZ-2** Compounds **1** (1.186 g, 2.5 mmol) and **3** (0.657 g, 1.0 mmol), (PPh_3_)_4_Pd(0) (2 mg, 0.0017 mmol), and a few drops of Aliquat 336 were dissolved in a mixture of toluene (30 mL) and aqueous 2 M Na_2_CO_3_ (4 mL). The solution was refluxed at 115 °C for 18 h under N_2_ atmosphere. The reaction mixture was cooled to RT, and the mixture was concentrated. The crude product was purified by column chromatography (silica gel, hexane/ethyl acetate = 1:5) to afford 0.966 g of **BTZCZ-2** (81%). ^1^H NMR (CDCl_3_, ppm): ™ 8.20 (d, 4H), 7.81 (d, 2H), 7.73 (m, 6H), 7.60 (m, 4H), 7.41 (m, 4H), 4.53 (t, 2H), 4.65 (m, 2H), 1.70 (m, 10H), 1.28–1.33 (m, 106H), 0.92 (t, 15H). ^13^C NMR (CDCl_3_, ppm) ™ 148.9, 145.0, 137.1, 136.5, 128.6, 128.0, 126.4, 124.9, 122.6, 121.5, 119.1, 117.2, 111.6, 109.6, 68.1, 63.4, 35.3, 31.9, 29.6, 29.3, 27.0, 26.7, 25.7, 22.7, 14.1. T_g_ = 51 °C, T_d_ = 365 °C.

**PBTZCZ-L** Compounds **2** (0.553 g, 1.0 mmol) and **3** (0.657 g, 1.0 mmol), (PPh_3_)_4_Pd(0) (2 mg, 0.0017 mmol), and a few drops of Aliquat 336 were dissolved in a mixture of toluene (20 mL) and aqueous 2 M Na_2_CO_3_ (2 mL). The solution was refluxed at 115 °C for 12 h under N_2_ atmosphere. To perform end-capping of the polymers, a small amount of bromobenzene was added first and stirred for 8 h to remove the boronic ester end group. Phenylboronic acid was then added, and the solution was stirred for another 8 h to remove the bromine end group. The reaction mixture was cooled to RT, and the mixture was slowly added to vigorously stirred methanol (100 mL) to obtain the polymer precipitate. The polymer was dissolved in chloroform and was passed through a short silica gel column. The filtrate was concentrated, re-precipitated in methanol, and finally purified by Soxhlet extraction using methanol and hexane. The hexane fraction was concentrated, filtered through a 0.45 μm Teflon filter, and precipitated in methanol to afford 0.463 g of **PBTZCZ-L** (57%). ^1^H NMR (CDCl_3_, ppm): ™ 8.22–8.17 (br, 4H), 7.90–7.53 (br, 8H), 4.90 (br, 2H), 4.68 (br, 1H), 2.50–1.80 (br, 6H), 1.50–0.78 (br, 45H). M_n_ = 3,500 g∙mol^−1^, PDI = 1.9. T_g_ = 98 °C, T_d_ = 415 °C.

**PBTZCZ-H** In order to afford the high-molecular-weight polymer **PBTZCZ-H**, the reaction was allowed to proceed for a long duration of 72 h using a procedure similar to that described for the polymer **PBTZCZ-L**. Due to its lower solubility than that of **PBTZCZ-L**, it can be further purified by subsequent Soxhlet extraction using acetone, hexane, and chloroform. The chloroform fraction was concentrated, filtered through a 0.45 μm Teflon filter, and precipitated in methanol to afford 0.568 g of **PBTZCZ-H** (70%). ™ 8.22–8.17 (m, 4H), 7.90–7.53 (br, 8H), 4.90 (m, 2H), 4.68 (m, 1H), 2.50–1.80 (m, 6H), 1.50–0.78 (br, 45H). M_n_ = 26,700 g∙mol^−1^, PDI = 5.6. T_g_ = 126 °C, T_d_ = 439 °C.

**Fabrication and characterization of solar cells** Photovoltaic properties were measured using the conventional device structure of indium-tin-oxide (ITO)/poly(3,4-ethylenedioxy-thiophene):polystyrene sulfonate (PEDOT:PSS)/active layer/LiF/Al and the inverted device structure of ITO/ZnO:polyethylenimine ethoxylated (PEIE)/active layer/MoO_3_/Ag. ITO substrates were cleaned with detergents, DI water, acetone, and isopropyl alcohol by sonication for 20 min each. The substrates were then treated with O_2_ plasma for 10 min. For the conventional device, a hole-transporting layer of PEDOT:PSS (Baytron PVP Al 4083) was spin-coated at 4000 rpm for 30 s and annealed at 120 °C for 1 h under N_2_ atmosphere. Active layers were deposited on the PEDOT:PSS layer by spin casting at 2000 rpm for 60 s from a 1,2-diclorobenzene solution containing 2.0 wt% of BTZ-based materials:PC_71_BM (1:2 *w*/*w*). The samples were annealed at 80 °C for 10 min prior to thermal evaporation of LiF (0.8 nm) and Al (100 nm). For the inverted devices, the ZnO solution was spin-coated at 4000 rpm onto ITO glass and annealed at 200 °C for 10 min in ambient atmosphere. After spin-coating the active layer, MoO_3_ (10 nm) and Ag (100 nm) were subsequently evaporated under high vacuum (<1 × 10^−4^ Pa). The dark and illuminated *J-V* characteristics of the solar cells were tested using a Keithley 2400 source meter and an Oriel white light source under AM 1.5, illuminated at 100 mW/cm^2^.

**Synchrotron X-ray scattering measurements** Synchrotron X-ray scattering measurements were carried out at the 3C beamline of the Pohang Accelerator Laboratory in Korea. The X-ray wavelength was 1.1651 Å. Two-dimensional X-ray patterns were recorded using a CCD (Rayonix 2D SX165) X-ray detector. The distance between the sample and the detector was 50 cm, and the exposure time was 20 s. The samples in the powder state were held for at least 10 min at the measurement temperature, and then X-ray scattering measurements were recorded.

## 3. Results and Discussion

**Scheme 1:**
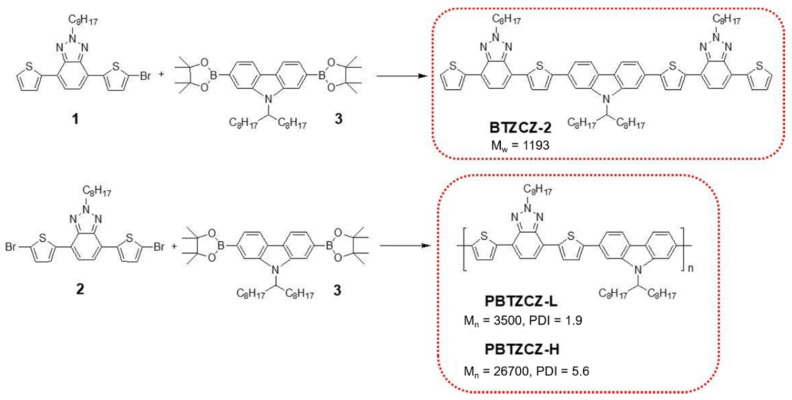
The synthesis of small molecule (**BTZCZ-2**), oligomer (**PBTZCZ-L**), and high-molecular-weight polymer (**PBTZCZ-H**) containing BTZ building blocks.

Scheme 1 shows the synthesis of BTZ-based small molecule (**BTZCZ-2**), low-molecular-weight oligomer (**PBTZCZ-L**), and high-molecular-weight polymer (**PBTZCZ-H**). The starting compounds were prepared as previously described [22,23,24], and their chemical structures were confirmed by ^1^H NMR and ^13^C NMR spectroscopy. **BTZCZ-2**, **PBTZCZ-L**, and **PBTZCZ-H** were prepared by Pd(0)-catalyzed Suzuki coupling reactions with carefully controlled molar ratios of the reactants. They were soluble in common organic solvents such as chloroform, toluene, chlorobenzene, and tetrahydrofuran owing to the long alkyl chain at the 2-position on the BTZ units. Notably, **BTZCZ-2** and **PBTZCZ-L** were soluble even in acetone and hexane, whereas **PBTZCZ-H**, a high-molecular-weight polymer, was not. Therefore, after removing low-molecular-weight oligomers by subsequent Soxhlet extraction using acetone and hexane, we obtained **PBTZCZ-H** with a high number-average molecular weight (M_n_). Table 1 lists the physical properties, including the molecular weights, of the polymers. The M_n_ values of **PBTZCZ-L** and **PBTZCZ-H** were 3500 and 26,700 g∙mol^−1^, respectively, and their PDIs were 1.9 and 5.6, respectively.

**Figure 1:**
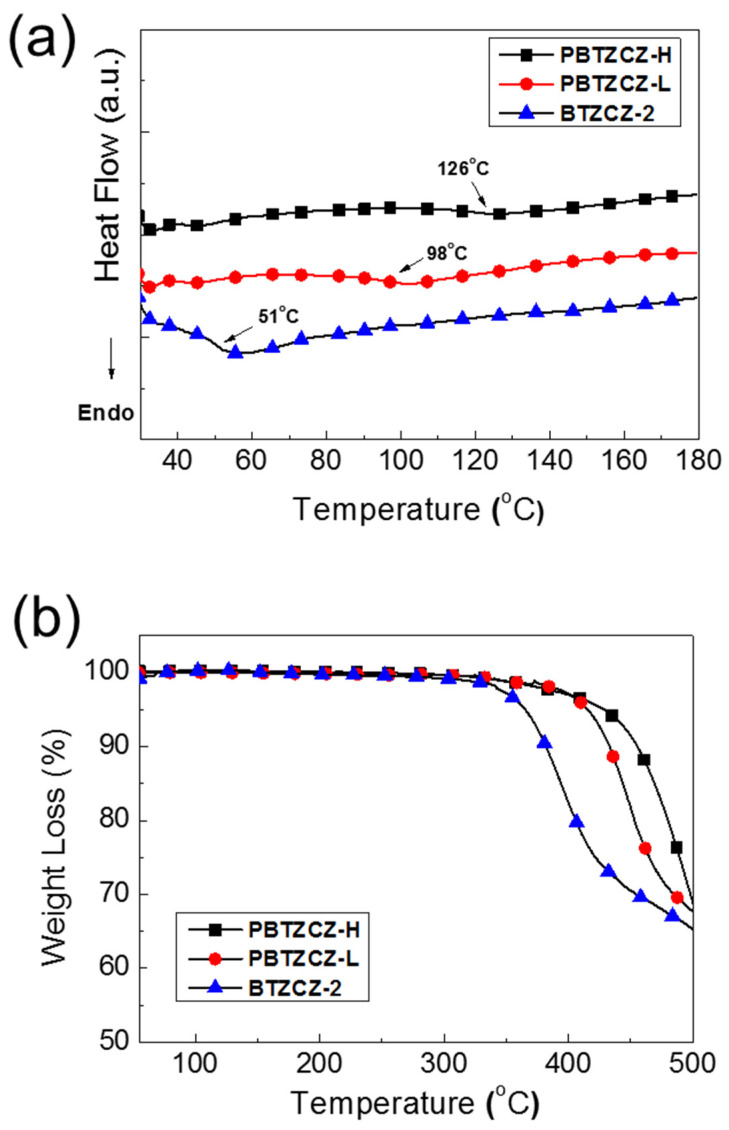
(**a**) DSC and (**b**) TGA data for **BTZCZ-2**, **PBTZCZ-L**, and **PBTZCZ-H** samples showing thermal transition and thermal stability.

Figure 1 shows the DSC and TGA results of **BTZCZ-2**, **PBTZCZ-L**, and **PBTZCZ-H** at a ramping rate of 10 °C/min under N_2_ atmosphere. As seen in Figure 1a, the glass transition temperature (T_g_) of the small molecule **BTZCZ-2** was observed ~51 °C, but the melting temperature was not observed due to the flexible alkyl groups. The T_g_ values of **PBTZCZ-L** and **PBTZCZ-H** were 98 °C and 126 °C, respectively. They showed good thermal stability, indicating that the initial decomposition temperatures (5% weight loss, T_d_) were found to be 365 °C, 415 °C, and 439 °C for **BTZCZ-2**, **PBTZCZ-L**, and **PBTZCZ-H**, respectively, as shown in Figure 1b.

**Figure 2:**
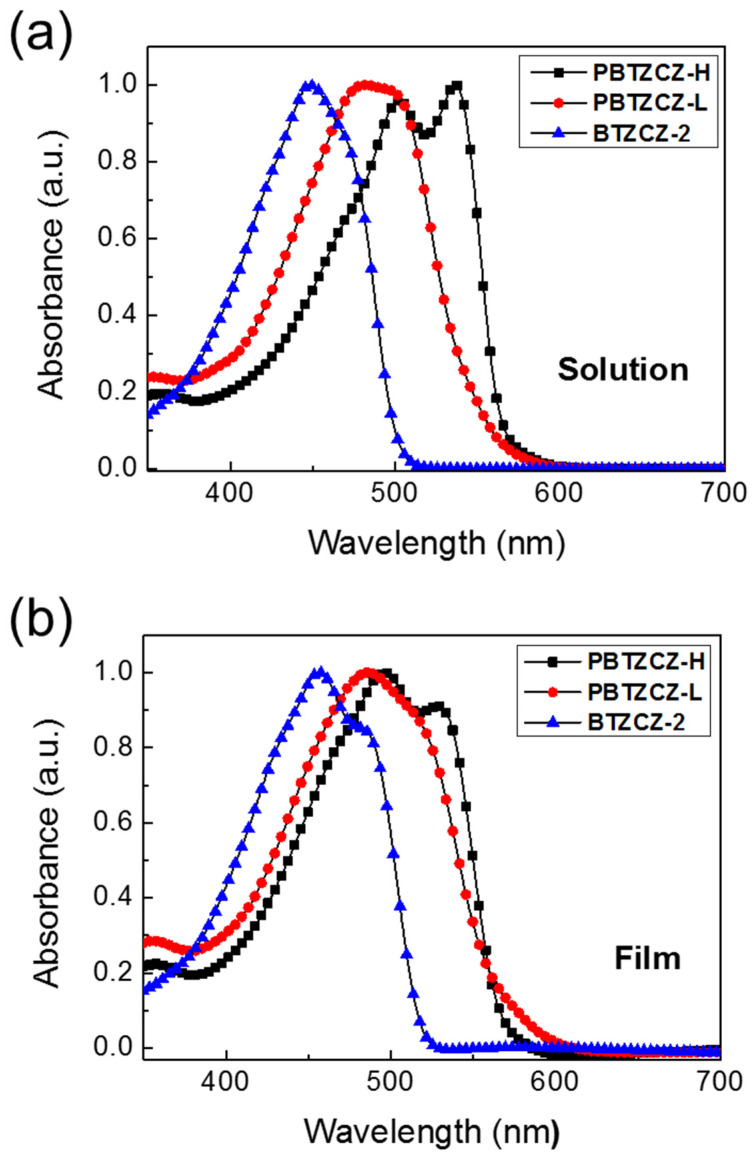
UV/vis absorption spectra of **BTZCZ-2**, **PBTZCZ-L**, and **PBTZCZ-H** (**a**) in chlorobenzene solution and (**b**) as thin solid films.

Figure 2 shows the UV/vis absorption spectra of **BTZCZ-2**, **PBTZCZ-L**, and **PBTZCZ-H** in solution and as thin solid films. In dilute chlorobenzene solution, the absorption maxima of **BTZCZ-2**, **PBTZCZ-L**, and **PBTZCZ-H** were located at 449, 481, and 536 nm, respectively, caused by the intramolecular charge transfer between the BTZ and carbazole units. **PBTZCZ-H** exhibited the broadest absorption bands between 400 and 600 nm, with two absorption maxima peaks at 502 nm and 536 nm. Compared to that of **PBTZCZ-H** as a solution, the absorption spectrum of **PBTZCZ-H** as a thin solid film remained almost unchanged. However, **BTZCZ-2** and **PBTZCZ-L** as thin solid films exhibited a slight bathochromic shift (~5 nm) compared to their spectra recorded in the solution form, which might be due to the aggregation in the solid state.

Moreover, the cut-off absorption wavelength for **PBTZCZ-L** was estimated to be ~600 nm, which was higher than that of **PBTZCZ-H** (585 nm), even though the absorption maximum value for **PBTZCZ-L** was shown to be lower than that of **PBTZCZ-H**. These results suggest that small molecules and oligomers based on BTZ building blocks with carbazole units have better intermolecular interactions and aggregation in the solid state compared to those of high-molecular-weight polymers. The optical band gap (E_g_) values, calculated from the cut-off absorption wavelength of the solid-state films, were 2.34 eV for **BTZCZ-2**, 2.11 eV for **PBTZCZ-L**, and 2.12 eV for **PBTZCZ-H**.

**Figure 3:**
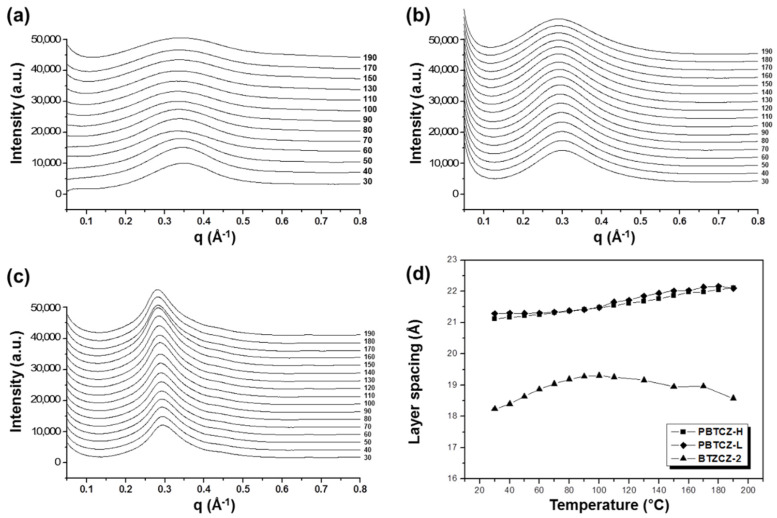
X-ray scattering profiles for (**a**) **BTZCZ-2**, (**b**) **PBTZCZ-L**, and (**c**) **PBTZCZ-H** at 30–190 °C, and (**d**) dependence of layer spacing of the samples on increased temperature.

The X-ray scattering data for **BTZCZ-2**, **PBTZCZ-L**, and **PBTZCZ-H** during the heating process are shown in Figure 3. The structural changes occurring during the first heating, which were unstable during sample preparation, are not shown here. A chain rearrangement occurred during the second heating. It should be noted that the peak of **PBTZCZ-H** with a high molecular weight was located at the lowest q value. The q value is associated with the layer spacing between the polymer molecules. As shown in Figure 3d, the layer spacing of the polymers is dependent on the temperature change. The spacing for **BTZCZ-2** was the shortest, ranging from 18 to 19 Å. It increased rapidly and then gradually decreased with increasing temperature. On the other hand, those for **PBTZCZ-L** and **PBTZCZ-H** only tended to increase up to 22 Å. This tendency is related to the intermolecular bonding forces at elevated temperatures. Because the intermolecular force is small in **BTZCZ-2** despite the relatively narrow gap between the molecules (in comparison with that seen in other two specimens), the structure changed sensitively with temperature. The structures of the polymers changed smoothly owing to the intermolecular bonding of long chains with high molecular weights. 

**Figure 4:**
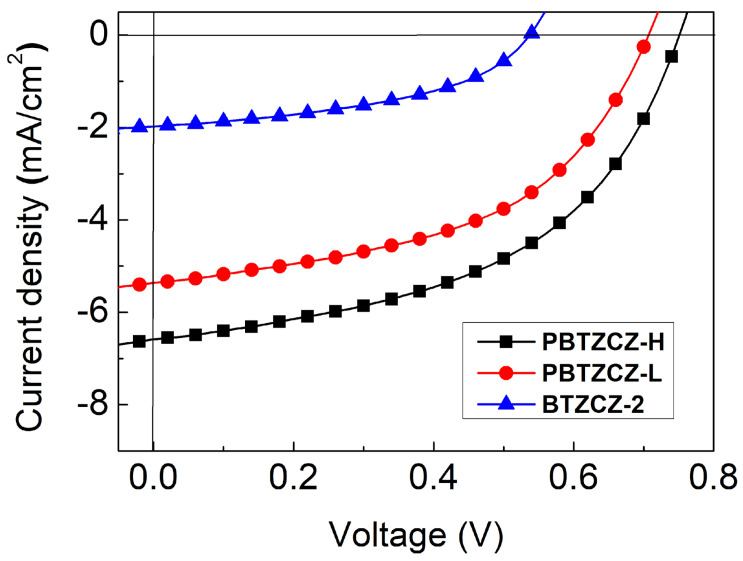
*J-V* characteristics of the **BTZCZ-2**-, **PBTZCZ-L**-, and **PBTZCZ-H**-based solar cell devices.

Photovoltaic properties were measured using the conventional (ITO/PEDOT: PSS/active layer/LiF/Al) and inverted (ITO/ZnO:PEIE(1:2)/active layer/MoO_3_/Ag) device structures. The active layers of the solar cells were fabricated by spin-casting a 1:2 (*w*/*w*) mixture of BTZ-based materials with PC_71_BM. After spin coating, the films were thermally annealed at 80 °C for 10 min. The corresponding *J-V* curves for conventional device structures under simulated AM 1.5G solar irradiation (100 mW/cm^2^) are shown in Figure 4, and the detailed device parameters are summarized in Table 2. The **BTZCZ-2**:PC_71_BM-based device showed low device efficiency, with PCE of 0.49%, *J*_sc_ of 1.97 mA/cm^2^, *V*_oc_ of 0.54 V, and FF of 45.9%. Device made from an oligomer type of **PBTZCZ-L** showed better device performance: PCE = 1.87% (*J*_sc_: 5.36 mA/cm^2^, *V*_oc_: 0.71 V, and FF: 49.5%), which was almost four times higher than that of the **BTZCZ-2**-based device. The enhanced PCE was attributed to the lower optical band gap and broader absorption spectrum

The best solar cell performance in the conventional device structures was obtained using **PBTZCZ-H**. Its PCE was 2.43% with *J*_sc_ of 6.58 mA/cm^2^, *V*_oc_ of 0.74 V, and FF of 49.2%. While the *J_sc_* of the **PBTZCZ-H-based** device significantly increased by over 20%, the *V*_oc_ barely increased by less than 5% compared to that of the **PBTZCZ-L**-based device. These results indicate that **PBTZCZ-L** with M_n_ of 3500 g∙mol^−1^ already reached the effective conjugated length for the energy band gap, resulting in a *V_oc_* similar to that of the high-molecular-polymer **PBTZCZ-H**. On the other hand, the enhanced *J*_sc_ for the **PBTZCZ-H**-based device is explained by the efficient exciton dissociation and charge collection from the **PBTZCZ-H** and PC_71_BM domains, which increase with increasing molecular weight.

**Figure 5:**
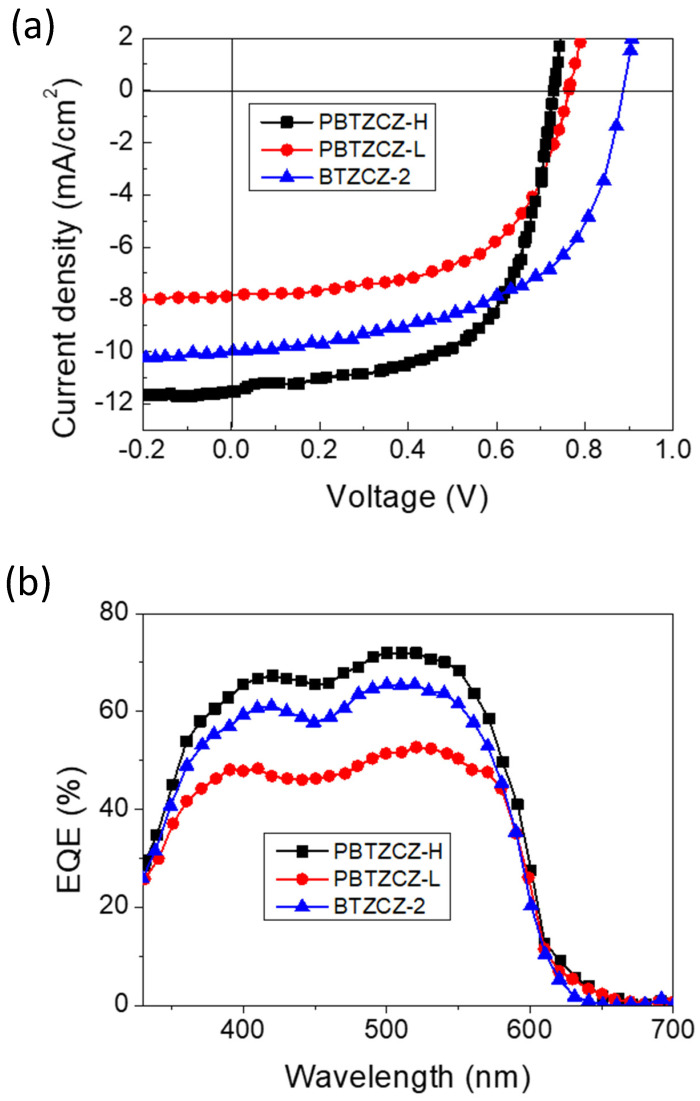
(**a**) *J-V* characteristics and (**b**) External quantum efficiency characteristics of the **BTZCZ-2**-, **PBTZCZ-L**-, and **PBTZCZ-H**-based inverted OPV devices.

We found that these active materials showed improved device performance with inverted device structures with two different interfacial layers (ZnO only and a combination of ZnO and PEIE). It is known that the polyelectrolyte PEIE introduced on top of ZnO, which is used as the electron-collecting interlayer, can improve the electron extraction and transport efficiency at the cathode in inverted OPV devices [25,26]. **PBTZCZ-H-** and **PBTZCZ-L**-based devices with only ZnO layer showed PCEs of 3.81% and 3.53%, respectively, and high *J_sc_*, *V_oc_*, and FF. Surprisingly, the device performance of **BTZCZ-2** was similar to that of **PBTZCZ-L** and **PBTZCZ-H**; The PCE was 3.76% with *J*_sc_ of 7.71 mA/cm^2^, *V*_oc_ of 0.87 V, and FF of 56.5%. Further enhancement of the photovoltaic performance was achieved by using a combination of ZnO and PEIE layers (1:2). The **PBTZCZ-H**-based device showed the highest PCE of 5.13% with *J*_sc_ of 11.5 mA/cm^2^, *V*_oc_ of 0.74 V, and FF of 60.1%. The **BTZCZ-2**-based device showed a slightly lower PCE of 5.05%. This improvement is attributed to the reduced work function of the combined ZnO and PEIE (3.8 eV from 4.5 eV), resulting in an increase in the work function difference between the anode and cathode, thus leading to an increase in the *J_sc_*, *V_oc_*, and FF. Figure 5a shows the *J-V* curves of **BTZCZ-2**-, **PBTZCZ-L**-, and **PBTZCZ-H**-based devices. The external quantum efficiency (EQE) spectra of these devices are shown in Figure 5b. The EQE values of **BTZCZ-2**- and **PBTZCZ-H**-based devices reached 70% over a wide absorption range.

**Figure 6:**
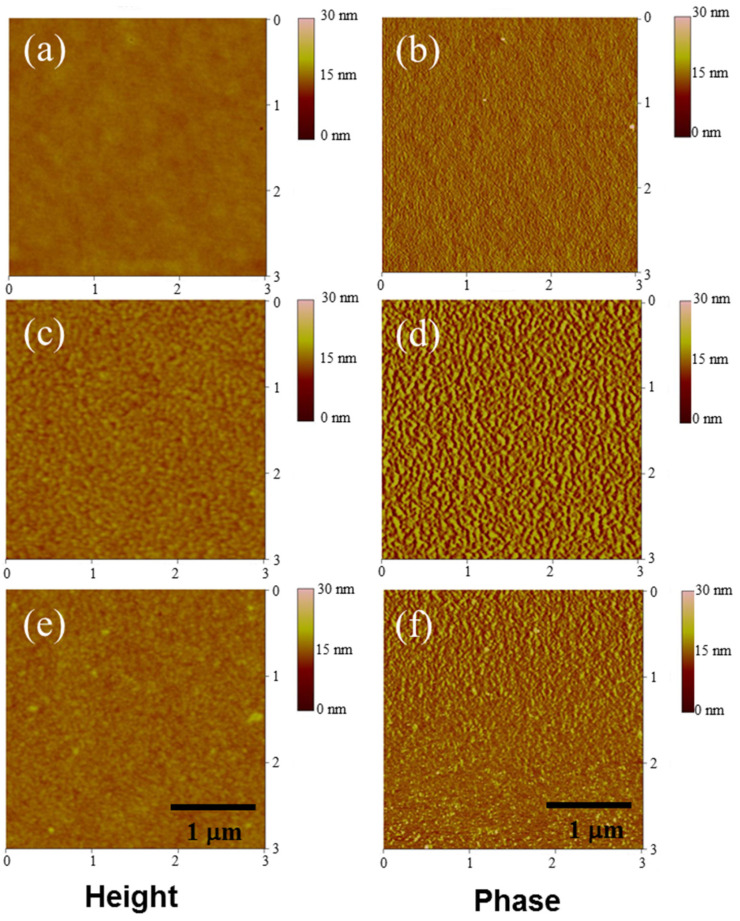
AFM images of the three active layer films in a 3 × 3 μm scale. Height images (**a**,**c**,**e**) and phase images (**b**,**d**,**f**) of **BTZCZ-2**, **PBTZCZ-L**, **PBTZCZ-H** based films.

The high PCE and *V*_oc_ of the **BTZCZ-2**-based device may originate from its superior film morphology compared to those of the **PBTZCZ-H**- and **PBTZCZL**-based devices. Polymer chains generally have stronger van der Waals interactions than do small molecules, causing more phase separation between the donor materials and the PC_71_BM. These different morphologies were characterized using tapping-mode AFM. Figure 6 shows the topographic height and phase images (3 × 3 μm). Notably, the small molecule-based **BTZCZ-2**:PC_71_BM film exhibited a more uniform surface than did the other two films. The root mean square (RMS) roughness values of **BTZCZ-2**:PC_71_BM, **PBTZCZ-L**:PC_71_BM, and **PBTZCZ-H**:PC_71_BM films were 0.36, 1.00, and 0.62 nm, respectively. 

## 4. Conclusions

We successfully synthesized D-A π-conjugated small molecule, oligomer, and polymer consisting of a BTZ building block as an acceptor and a carbazole unit as a donor. All materials showed good solubility and high thermal stability. The high-molecular-weight polymer **PBTZCZ-H** exhibited an M_n_ value of 26,700 g∙mol^−1^ and a medium optical band gap of 2.12 eV. A solar cell device using **PBTZCZ-H**:PC_71_BM possessed a PCE of 2.43% with *J*_sc_ of 6.58 mA/cm^2^, *V*_oc_ of 0.74 V, and FF of 49.2%. Further enhancement of the photovoltaic performance was achieved by using a combination of ZnO and PEIE layers (1:2). The **PBTZCZ-H-based** device showed the highest PCE of 5.13% at *J*_sc_ of 11.5 mA/cm^2^, *V*_oc_ of 0.74 V, and FF of 60.1%. The **BTZCZ-2**-based inverted solar cell device displayed a PCE of 5.05%, which is considerably higher than those obtained for conventional solar cells.

## Figures and Tables

**Table 1:** Physical properties of the **BTZCZ-2**, **PBTZCZ-L**, and **PBTZCZ-H**.

Sample	M_n_^a^ (g mol^−1^)	PDI ^b^ (M_w_/M_n_)	λ_abs_^c^ (nm)	λ_abs_^d^ (nm)	E_g_^e^ (eV)	T_g_^f^ (°C)	T_d_^g^ (°C)
BTZCZ-2	-	-	449	456	2.34	51	365
PBTZCZ-L	3500	1.9	481	486	2.11	98	415
PBTZCZ-H	26700	5.6	502, 536	503, 536	2.12	126	439

^a,b^ Determined by GPC with polystyrene as a standard and chlorobenzene as eluent; number-average molecular weight (M_n_), weight-average molecular weight (M_w_), and polydispersity index (PDI = M_w_/M_n_). ^c^ The maximum absorption wavelength measured in chlorobenzene solution. ^d^ The maximum absorption wavelength measured in a thin solid film on a glass substrate. ^e^ Optical energy band gap determined by the absorption onset of the films. ^f^ The glass transition temperature of the polymer measured by DSC. ^g^ The initial decomposition temperature of the polymer measured by TGA.

**Table 2:** Photovoltaic performances of the conventional and inverted devices based on the **PBTZCZ-H**, **PBTZCZ-L**, and **BTZCZ-2** with PC_71_BM (1:2).

Donors	Device Configuration	Interfacial Layer	*J_sc_* (mA/cm^2^)	*V_oc_* (V)	FF (%)	PCE (%)
**BTZCZ-2**	Conventional	-	1.97	0.54	45.9	0.49
	Inverted	ZnO	7.71	0.87	56.5	3.76
	Inverted	ZnO:PEIE	9.95	0.89	57.0	5.05
**PBTZCZ-L**	Conventional	-	5.36	0.71	49.5	1.87
	Inverted	ZnO	7.80	0.77	58.6	3.53
	Inverted	ZnO:PEIE	7.80	0.77	58.2	3.48
**PBTZCZ-H**	Conventional	-	6.58	0.75	49.2	2.43
	Inverted	ZnO	7.69	0.88	55.9	3.81
	Inverted	ZnO:PEIE	11.5	0.74	60.1	5.13

## Data Availability

The data presented in this study are available on request from the corresponding author.
